# Small cholangiolocellular carcinoma that was difficult to distinguish from cholangiocellular carcinoma: a case report

**DOI:** 10.1186/s40792-017-0377-0

**Published:** 2017-09-15

**Authors:** Norihiro Ishii, Kenichiro Araki, Takahiro Yamanaka, Tadashi Handa, Mariko Tsukagoshi, Takamichi Igarashi, Akira Watanabe, Norio Kubo, Shinichi Aishima, Hiroyuki Kuwano, Ken Shirabe

**Affiliations:** 1 Department of Hepatobiliary and Pancreatic Surgery, Graduate School of Medicine, Gunma University, 3-39-22, Showamachi, Maebashi, 371-8511 Japan; 2 Department of General Surgical Science, Graduate School of Medicine, Gunma University, 3-39-22, Showamachi, Maebashi, 371-8511 Japan; 3 Department of Diagnostic Pathology, Graduate School of Medicine, Gunma University, 3-39-22, Showamachi, Maebashi, 371-8511 Japan; 4 Department of Pathology and Microbiology, Faculty of Medicine, Saga University, 5-1-1, Nabeshima, Saga, 849-8501 Japan

**Keywords:** Atypical, Inflammatory cell, Doubling time, Hepatic progenitor cell, Slow growth

## Abstract

**Background:**

Cholangiolocellular carcinoma (CoCC) is thought to be derived from hepatic progenitor cells. Because of its origin, CoCC has diverse clinicopathological and imaging findings. Here, we report a case of small CoCC that was difficult to diagnose preoperatively.

**Case presentation:**

A 62-year-old woman was confirmed with a small liver nodule in the left lobe 2 years after a sustained virological response of hepatitis C virus. The size of the nodule was 11.9 × 6.1 mm, and 6 months later, the size increased to 12.5 × 7.8 mm. The doubling time of this tumor was 285 days. The tumor revealed peripheral early enhancement and delayed internal staining in dynamic computed tomography images and marked high intensity in diffusion-weighted magnetic resonance imaging scans. These imaging findings resembled those of cholangiocellular carcinoma (CCC). The tumor was removed by laparoscopic lateral sectionectomy. Pathological findings revealed that the tumor was composed of small cuboidal cells and showed irregular anastomosis small grand. Immunohistochemical findings showed that the tumor cells were negative for Hep-par 1 and positive for cytokeratin 19. Epithelial membrane antigen staining was positive for the membranous side of the lumen. According to these pathological findings, the tumor was diagnosed as CoCC.

**Conclusion:**

Although some characteristic imaging findings are reported for CoCC, they are not specific because of the variety in pathological findings. Especially, small CoCCs might have poor characteristic imaging findings and may be difficult to distinguish from CCC in the images. However, slow tumor growth might be one of the characteristics to suspect the possibility of a CoCC.

## Background

Cholangiolocellular carcinoma (CoCC) is rare and one of the primary malignant liver tumors, which is thought to originate from hepatic progenitor cells (HPCs) existing in the canals of Hering [1, 2]. Because of its origin, CoCC has diverse clinicopathological and imaging findings [3–5]. Recent advances in the study of correlations between image and pathological findings proposed that CoCCs have the dual imaging features of two major liver malignant tumors, hepatocellular carcinoma (HCC) and cholangiocellular carcinoma (CCC), according to the cellularity and amount of fibrous stroma [6]. Therefore, to reflect the diversity and its rarity, CoCCs are difficult to diagnose preoperatively [7] and are usually diagnosed from postoperative pathological findings.

Here, we report a case of a small CoCC after a sustained virological response (SVR) of hepatitis C virus (HCV), which was difficult to diagnose preoperatively. Although there are some characteristic imaging findings in CoCC, these findings are not specific because of the variety in pathological findings. Thus, when the tumor has poor characteristic imaging findings, preoperative diagnosis of CoCC is difficult.

## Case presentation

A 62-year-old woman was confirmed to have liver dysfunction due to HCV in other hospitals and was referred to our hospital for the treatment of HCV. Here, she was treated by interferon/ribavirin therapy for 24 weeks and achieved a SVR. Two years after SVR, a liver nodule that was not pointed out previously was detected in routine abdominal ultrasonography, and the size of the nodule was 11.9 × 6.1 mm. Six months later, the nodule size grew to 12.5 × 7.8 mm, as detected using ultrasonography. Laboratory data on liver function were nearly normal, and the levels of tumor markers such as alpha-fetoprotein, prothrombin induced by vitamin K absence or antagonist II, carcinoembryonic antigen, and carbohydrate antigen 19-9 were also normal. HCV-RNA remained negative.

**Fig. 1:**
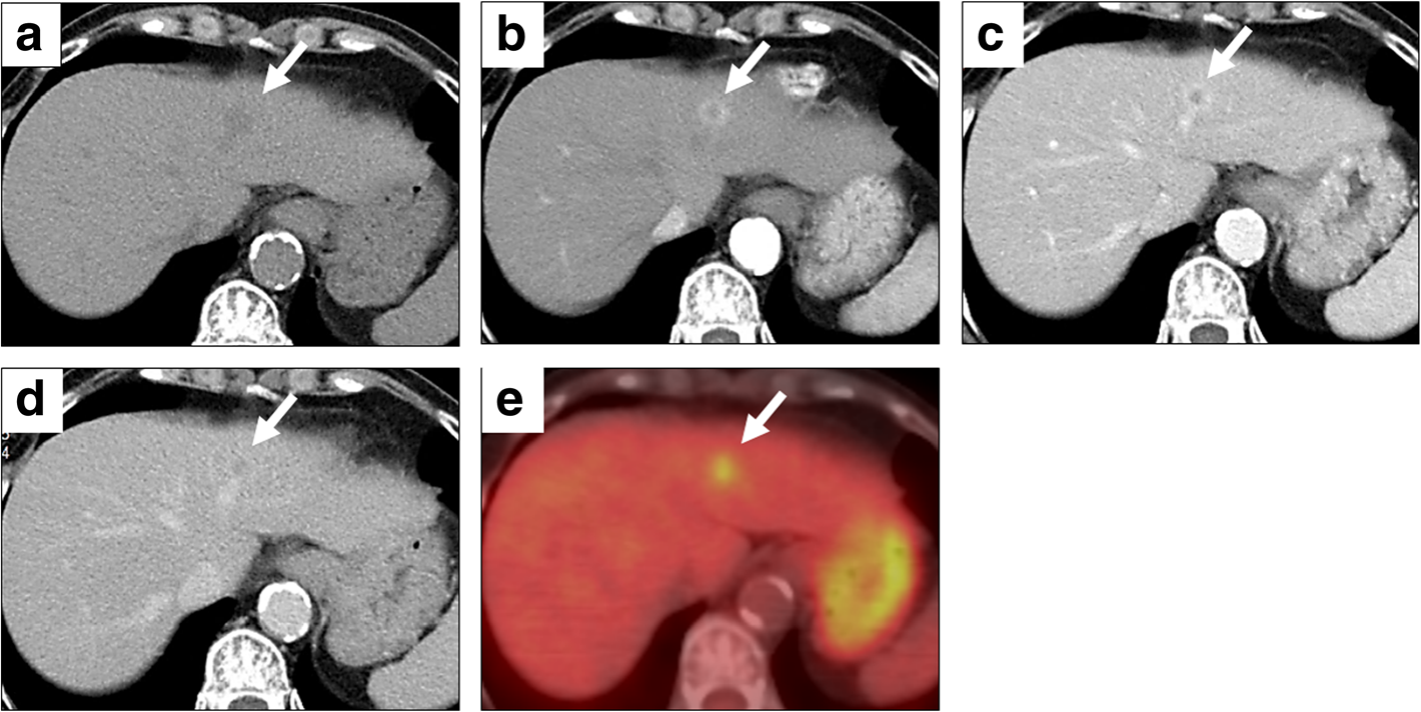
Findings of dynamic computed tomography (CT) and FDG-PET. **a** Conventional CT shows a low-density tumor whose margin is unclear in the left lobe of the liver. **b** The tumor shows marked enhancement at the periphery in the arterial phase. **c** The tumor shows prolonged enhancement at the periphery in the portal phase. **d** In late phase, the tumor shows slightly lower density relative to the normal liver but has a faint enhancement inside the tumor. **e** The tumor exhibits uptake of FDG, whose SUV max is 4.7

Computed tomography (CT) showed a small tumor in the left lobe of the liver. The tumor size was approximately 1.5 cm in diameter (Fig. 1), and the tumor showed low density on conventional CT (Fig. 1a). Dynamic CT revealed early marked enhancement at the periphery of the tumor (Fig. 1b). From portal to late phase, the tumor showed prolonged enhancement at the periphery and gradual enhancement inside the tumor, revealing slightly lower density relative to the normal parenchyma (Fig. 1c, d). Furthermore, 18F-fluorodeoxyglucose positron emission tomography (FDG-PET) showed FDG accumulation inside the tumor and the standardized uptake value maximum (SUV max) was 4.7. Capsule formation and vessel penetration within the tumor were not evident. In addition, there were no findings of lymph node swelling and distant metastasis.

**Fig. 2:**
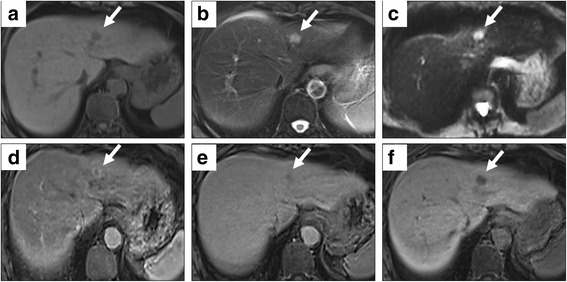
Findings of magnetic resonance imaging (MRI). The tumor shows low intensity in T1-weighted image (**a**) and shows high intensity in T2-weighted image (**b**). The tumor exhibits marked high intensity on the diffusion-weighted image (**c**). Dynamic MRI shows peripheral enhancement of the tumor in the early phase (**d**) and low intensity relative to the normal liver in the late phase (**e**). In the hepatobiliary phase, the tumor exhibits defects in enhancement (**f**)

With magnetic resonance imaging (MRI), the tumor exhibited low and high intensities in T1- and T2-weighted images, respectively (Fig. 2a, b). On the other hand, diffusion-weighted (DW) imaging showed remarkably high intensity, and the apparent diffusion coefficient (ADC) value was 1.11 × 10^− 3^ mm^2^/s (*b* value = 1000 s/mm^2^) (Fig. 2c). Dynamic MRI using the contrast agent gadolinium-ethoxybenzyl-diethylenetriamine penta-acetic acid showed ringed enhancement in the early phase and became lower in intensity gradually relative to the normal parenchyma in the late phase similar to dynamic CT findings (Fig. 2d, e). The tumor exhibited defects in enhancement in the hepatobiliary phase (Fig. 2f).

On the basis of these findings, we considered that this hepatic nodule was a malignant tumor and diagnosed this tumor as a CCC or HCC that showed atypical imaging findings. Although we discussed the possibility of it being a metastatic liver tumor, there were no findings to suspect malignant tumors in other organs. Then, laparoscopic lateral sectionectomy of the liver was performed.

**Fig. 3:**
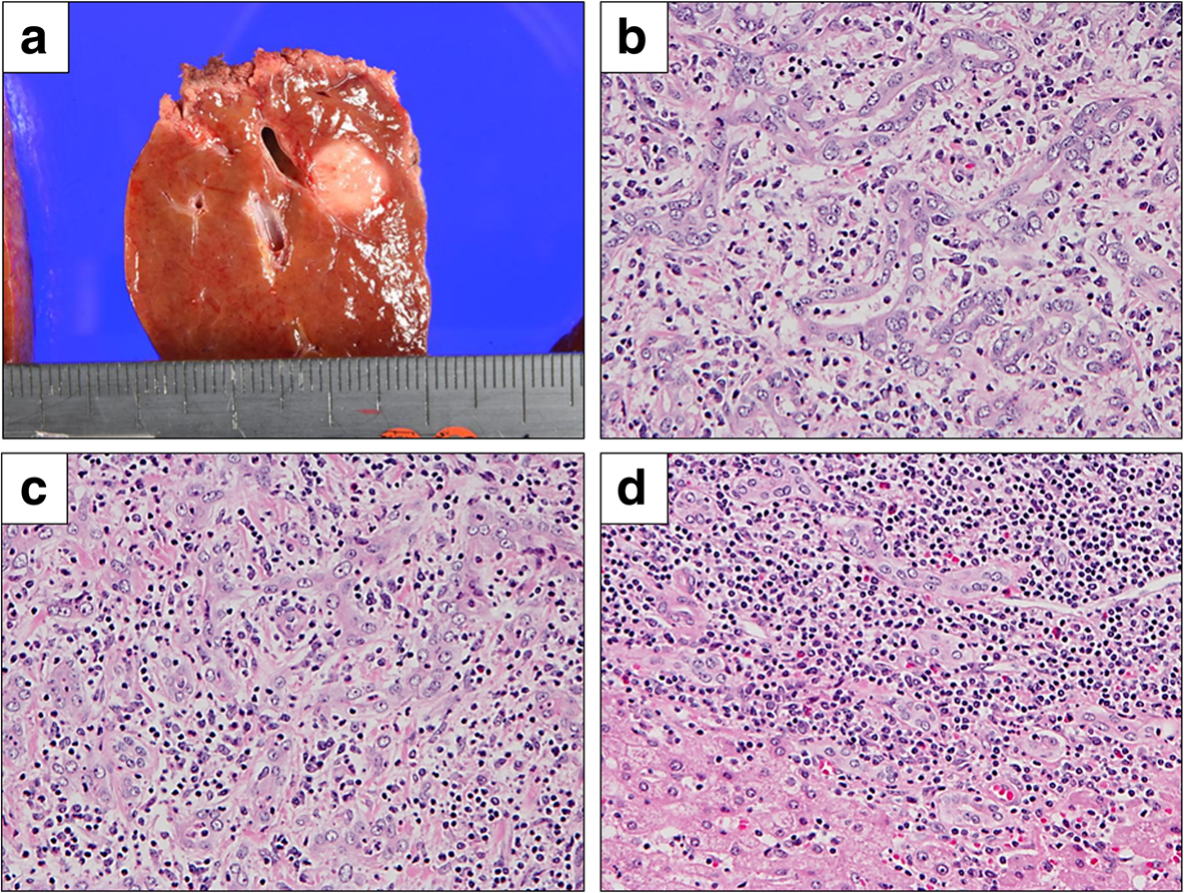
Macroscopic and histopathological findings of the tumor. **a** The tumor is white in color and the size of the tumor is 1.1 cm in diameter. **b** The tumor cells with enlarged nuclei form an irregular small gland with inflammatory stroma, suggesting adenocarcinoma. **c** The tumor cells with oval vesicular nuclei grow in cord-like or anastomosing branching patterns. **d** Tumor periphery shows dense inflammatory cells with ductular reaction. The cells and their nuclei of reactive ductules are smaller than those of adenocarcinoma. (**b**–**d** Original magnification × 200)

**Fig. 4:**
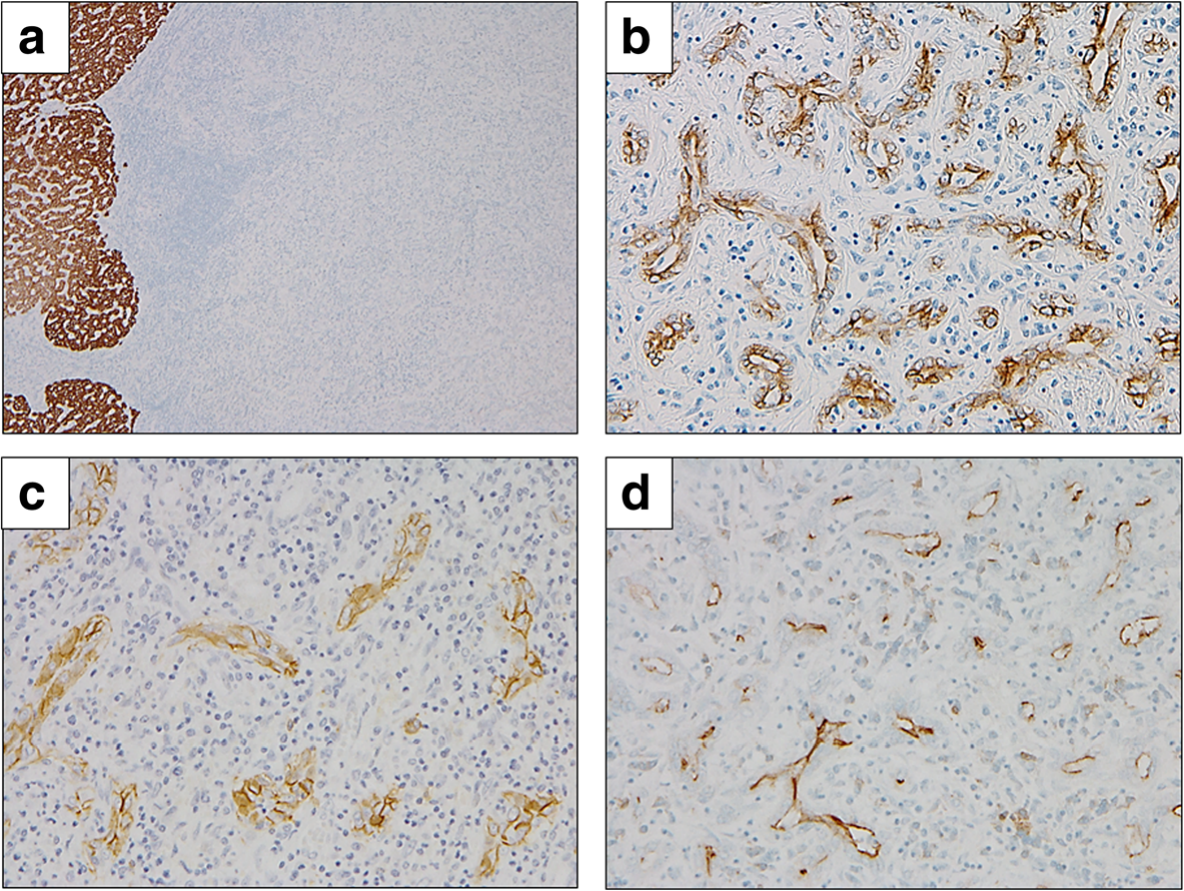
Immunohistochemical staining of the tumor. **a** The tumor cells are negative for Hep-par 1. Original magnification × 40. **b** The tumor cells arranged in irregular tubules with anastomosing pattern are positive for CK19. **c** The tumor cells show membranous stains for NCAM. **d** The luminal side of tumor cells are positive for EMA. (**b**–**d** Original magnification × 200)

The macroscopic findings of the tumor revealed a white color and a maximal diameter of 1.1 cm. Capsule formation was not observed (Fig. 3a). The tumor was composed of small cuboidal cells with clear nucleoli and showed irregular anastomosis small grand. The inflammatory cells showed remarkable infiltration, and vascular proliferation and ductular reactions were also seen at the peripheral lesion. (Fig. 3b–d). Immunohistochemical findings showed that the tumor cells were negative for Hep-par 1 (Fig. 4a) and positive for cytokeratin (CK) 19 and NCAM (Fig. 4b, c). Epithelial membrane antigen (EMA) staining was positive for the membranous side of the lumen (Fig. 4d). According to these pathological findings, the tumor was diagnosed as a CoCC. There were no events after operation, and the patient was discharged on postoperative day 8 and is alive without recurrence at the time of this report.

## Discussion

CoCC is a rare malignant liver tumor that has been categorized as a combined hepatocellular-cholangiocarcinoma with stem cell features, a cholangiolocellular subtype in the latest World Health Organization (WHO) classification. Recent studies revealed that CoCC derives from HPC, which has stem cell features and can differentiate into both hepatocytes and cholangiocytes [1, 3]. However, some researchers have proposed the possibility that CoCC derives from interlobular ducts, not canals of Hering or cholangioles in which HPCs exist, considering morphometric and immunohistochemical studies of CoCC [8, 9]. These reports revealed that the size of CoCC cancer ducts was far larger than that of the cholangioles and similar to that of the interlobular ducts. The mean diameters of the CoCC ducts, cholangioles, and interlobular ducts were 31.8, 13.8, and 26.5 μm, respectively [8]. Furthermore, the immunohistochemical staining patterns such as the membranous pattern of EMA and positivity for progenitor cell markers were seen not only in the cholangioles but also in the interlobular ducts. In fact, the cancer duct size in our case was almost larger than that of the cholangioles described above, and we cannot deny the possibility of interlobular duct origin based on the immunohistochemical staining. Thus, the origin of CoCC still remains controversial, and more detailed molecular studies might be needed to clarify the origin of CoCC.

Although the detailed mechanisms of CoCC carcinogenesis remain unclear, clinicopathological studies have revealed the presence of chronic liver injury by chronic viral hepatitis, non-alcoholic steatohepatitis, and alcoholism in CoCC patients [1, 10]. Additionally, previous reports revealed that HPCs were activated by chronic liver injury and formed ductular reactions [11, 12]. These findings have proposed that activation of HPCs by chronic liver injury is one of the etiologies of CoCCs. In fact, our case also had a prior infection by HCV before the diagnosis of the liver tumor. Although our case achieved a SVR, the history of HCV infection was considered to remain the risk of carcinogenesis of CoCC from chronic liver injury.

Reports of imaging findings of CoCC have been diverse [5, 6, 13, 14]. To reflect the characteristics of origin cells that have the potential to differentiate into both hepatocytes and cholangiocytes, CoCCs can show dual characteristics of HCCs and CCCs in images, such as whole early enhancement with delayed washout and peripheral early enhancement with centripetal filling, respectively. These findings are considered to depend on cellularity and the amount of fibrous stroma [6]. Additionally, CoCCs are considered to be comprised of various histologically characteristic areas, such as CoCC, HCC, and CCC areas in various proportions [1, 15]. Kozaka et al. [14] defined “pure CoCC” as a tumor that consists exclusively of CoCC without any HCC/CCC components. In this report, the characteristics of pure CoCC that were revealed in CT findings, compared with CCC, were hypervascularity, peritumoral enhancement in the arterial phase, the presence of intratumoral portal tracts, rare intrahepatic bile duct dilatation, and prolonged staining in the late phase. Some early enhancement of CoCC was considered to be derived from the high cellularity and tumor blood sinusoids. Non-pure CoCC showed intermediate findings between pure CoCC and CCC. Thus, these reports suggest that imaging findings of CoCC might be different by component proportions and may be difficult to diagnose preoperatively. In our case, the tumor size was very small, approximately 1.1 cm in diameter. Moreover, because of the inflammatory cell infiltration within the tumor, histological morphology was modified by inflammation; replacing growth of tumor cells is hardly observed due to the dense inflammatory cell infiltration at the tumor periphery. Nevertheless, typical pathological findings of CoCC such as small tumor cells with oval nuclei, growing in cord-like anastomosing patterns, were present. Furthermore, considering that immunohistochemical staining of EMA was positive for the membranous side of lumen and the tumor cells were negative for Hep-par 1, but positive for CK19 and NCAM, the diagnosis of CoCC seemed adequate. Although there were no obvious HCC and CCC areas within the tumor, we could not conclude whether our CoCC case was a “pure CoCC” or not due to the histological modification caused by the inflammatory cell infiltration. Imaging findings of our case such as peritumoral early enhancement, delayed internal staining, and the absence of bile duct dilatation were similar. However, these findings are not specific and are sometimes observed in CCC. Furthermore, other characteristics such as the intratumoral portal vein were considered being unclear due to its small size in our case.

The DW image on MRI was of high intensity in our case. The DW image represents the rate of diffusion of water molecules in tissues, and the ADC value is the quantitative value of DW image intensity. There were several reports mentioning that the ADC value was helpful for diagnosing and characterizing hepatic nodules, and ADC values of the hepatic malignant tumor were significantly lower than those of benign lesions [16, 17]. However, among subtypes of malignant tumors, the mean ADC values of CCC and HCC were reported as 0.95–1.01 × 10^− 3^ mm^2^/s and 0.90–1.12 × 10^−3^ mm^2^/s (*b* value = 1000 s/mm^2^), respectively [17, 18]. On the other hand, the ADC value of our CoCC case was 1.11 × 10^−3^ mm^2^/s (*b* value = 1000 s/mm^2^). Although there is no previous report to evaluate the ADC value of CoCC, these data suggest that it is difficult to distinguish CoCC from other liver malignant tumors using ADC values.

Reports regarding FDG uptake of CoCC with FDG-PET in English literature are very few, and only some case reports exist [7, 19]. The SUV max of our previous reported cases were 12.8 [7] and 25.2 [19], and of this present case was 4.7. Like this, although the SUV max of CoCC vary, the tumor sizes of both previous cases are larger than this present case. Thus, there is a possibility that the SUV max of CoCC depends on its tumor volume or size, similar to CCC [20]. However, accumulation of further analyses will be needed to conclude the significance of FDG uptake of CoCC.

CoCC is considered to have a favorable outcome after curative resection compared with CCC, and the median tumor size of CoCC is smaller than that of CCC [10]. This result suggests that the growth rate of CoCC may be relatively slow. In fact, the tumor doubling time (TDT) of our case was 285 days by calculation, as described previously [21]. On the other hand, median TDTs of HCC and CCC were reported to be 85.7 and 70 days, respectively. These data suggest that slow growth might be one of the characteristics of CoCC. This characteristic may be helpful for deciding upon the indication of liver transplantation in liver tumors showing imaging findings similar to those in CCC. Liver transplantation of CCC is not established because of its poor outcome after liver transplantation [22]. Although liver transplantation of CoCC is also not established, it may be selected as one of the treatments for CoCC in the future, because CoCC shows favorable outcomes after resection. Thus, when hepatic tumors reveal similar imaging findings to CCC, but also exhibit slow growth, we must consider the possibility of CoCC, and biopsy might be helpful for the decision of treatment strategy, especially in cases of liver transplantation.

In pathological findings of our case, prominent inflammatory cell infiltration was characteristic. To our knowledge, there are no reports describing marked inflammatory cell infiltration within CoCC. The reason why marked inflammatory cell infiltration occurred in our case remains unclear. However, recent studies have revealed that CoCC is a distinct molecular entity compared with other HPC-derived liver tumors, as determined by gene profiling analysis, and also revealed that CoCC shows significant upregulation of TGF-beta signaling and inflammatory and immune response signatures, such as interleukin-6, TNF-α, and chemokines and their receptors, which are known as factors of angiogenesis and inflammatory cell infiltration into the tumor [23]. These findings suggest that CoCC is likely to be closely related to angiogenesis and inflammation. If this hypothesis is correct, prominent inflammatory cell infiltration and vascular proliferation of our case can be explained. However, further study will be required to elucidate the significance of upregulation of such genes.

## Conclusions

We herein presented the case of a small CoCC that was difficult to diagnose preoperatively. There are some characteristic imaging findings in CoCC, but these are not specific because of the variety in pathological findings. Although it is possible to suspect CoCC when some characteristic imaging findings, such as hypervascularity, peritumoral enhancement in the arterial phase, presence of intratumoral portal tracts, absence of bile duct dilatation, and prolonged staining in the late phase, are combined, if they are not combined, the preoperative diagnosis of CoCC is considered rather difficult. Especially, small CoCCs might have poor characteristics of the abovementioned imaging findings and may be difficult to distinguish from CCC on imaging. However, slow tumor growth might be one of the characteristics to suspect the possibility of a CoCC. Recent advances are gradually clarifying the clinicopathological characteristics of CoCC and expected to progress the comprehension of CoCC.

## Abbreviations

ADC: Apparent diffusion coefficient
CCC: Cholangiocellular carcinoma
CK: Cytokeratin
CoCC: Cholangiolocellular carcinoma
CT: Computed tomography
DW: Diffusion-weighted
EMA: Epithelial membrane antigen
FDG-PET: 18F-fluorodeoxyglucose positron emission tomography
HCC: Hepatocellular carcinoma
HCV: Hepatitis C virus
HPC: Hepatic progenitor cell
MRI: Magnetic resonance imaging
SUV max: Standardized uptake value maximum
SVR: Sustained virological response
WHO: World Health Organization
